# 

**Published:** 1885-12-20

**Authors:** 


### TABLE OF CONTENTS.

ORIGINAL AND SELECTED ARTICLES:

Icteroid Fever, by B. F. Humphreys, M.D., Hawkins, Texas, 441. Epithelioma Treated with Arsenic, by J. B. Medlock, M. D., of Georgia, 448. Notes of Treatment by Prof. Da Costa, by the Reporter of "Class-Room Notes," 449. Successful Treatment of Lacerations and Fissures of the Os Uteri of Long Standing without Surgical Operation, by Bedford Brown, M.D., of Alexandria, Va., 450. Note on Hydro-Bromate of Hyoscine, by H. C. Wood, M. D., 455. Hopeine—(Alkaloid from Hops); Freckles, 456.

ABSTRACTS AND GLEANINGS:

Hypodermic Injections of Quinine in Malarial Fevers, 457. Hypodermic Injection of Morphine in Uremic Convulsions, 458. The Dangers of the Cocaine Habit, 459. Bi-hydrobromate of Quinia; Dover's Powder and its Modifications, 460. Creasote Water as a Local Anæsthesia, 461. Uses of Vinegar; Urethan—A New Hypnotic, 462. Hæmophilia; Successful Cerebral Surgery, 463. What is Murder; Treatment of Hemorrhoids, 464. Should Arsenic be Administered to Nursing Women; To Remove Lime or Chemical Substances from the Eye; Swallowing a Scarf-Pin, 465. Nephorotomy for Total Suppression of Urine; Some Uses of Cocaine in Gynecology; For Infantile Eczema, 466. Salicylic Acid and Castor Oil in Psoriasis; Some Lady Advice to Doctors; Cocaine in Cracked Nipples; Menthol, 467. Chaulmoorgra Oil in Chronic Squamous Eczema; Granules of Podophyllin and Hydrastin for Headache; Chloride of Calcium; Cocaine in Obstinate Vomiting, 468. A New Hemostatic Agent; Turpentine in Malignant Tumors; Water as a Local Anæsthetic; Swamp-Button-Bush in Rhus Poisoning; Gokhru; The Sims Memorial Fund, 469. A New Remedy for Hiccough; Cocaine in the Treatment of Whooping Cough; Anisic Acid; Vomiting of Pregnancy; Proprietary Remedies, 470.

SCIENTIFIC ITEMS:

The Pyrophore; A German, 471. Put Your Light in Front of the Horse; Paper in Tonkin, 472

PRACTICAL NOTES AND FORMULÆ.

Bromidia; Therapeutics of Cephalalgia, 473.

EDITORIALS AND MISCELLANEOUS.

Our Journal in 1886; Subscribers on Renewal; Prevention of Cholera; Our Premium Offer for 1886; Hymeneal; Vaccination; Hydrobromate of Caffein, 475. Gratuities of Physicians, 476. Atlanta Society of Medicine; The Medical News Visiting List; Our January (1886) Number, 477. Books and Pamphlets Received, 478. Receipted; Special Notices, 480.

